# Energy Loss in a MEMS Disk Resonator Gyroscope

**DOI:** 10.3390/mi10080493

**Published:** 2019-07-24

**Authors:** Jianbing Xie, Yongcun Hao, Weizheng Yuan

**Affiliations:** 1 School of Mechanical Engineering, Northwestern Polytechnical University, Xi’an 710072, China; 2 Key Laboratory of Micro/Nano Systems for Aerospace, Ministry of Education, Xi’an 710072, China

**Keywords:** disk resonator gyroscope (DRG), quality factor (Q), energy loss, thermoelastic damping, anchor loss, electronic damping

## Abstract

Analysing and minimizing energy loss is crucial for high performance disk resonator gyroscopes (DRGs). Generally, the primary energy loss mechanism for high vacuum packaged microelectromechanical system (MEMS) resonators includes thermoelastic damping, anchor loss, and electronic damping. In this paper, the thermoelastic damping, anchor loss, and electronic damping for our DRG design are calculated by combining finite element analysis and theoretical derivation. Thermoelastic damping is the dominant energy loss mechanism and contributes over 90% of the total dissipated energy. Benefiting from a symmetrical structure, the anchor loss is low and can be neglected. However, the electronic damping determined by the testing circuit contributes 2.6%–9.6% when the bias voltage increases from 10 V to 20 V, which has a considerable impact on the total quality factor (Q). For comparison, the gyroscope is fabricated and seal-packaged with a measured maximum Q range of 141k to 132k when the bias voltage varies. In conclusion, thermoelastic damping and electronic damping essentially determine the Q of the DRG. Thus, optimizing the resonance structure and testing the circuit to reduce energy loss is prioritized for a high-performance DRG design.

## 1. Introduction

Microelectromechanical system (MEMS) gyroscopes, which are used to detect rotation angle or angular velocity, have been researched and developed for 30 years. Since the first MEMS gyroscope was reported in 1988 [1], various MEMS gyroscopes, such as single-mass gyroscopes [2,3], tuning-fork gyroscopes [4,5], and vibratory-wheel gyroscopes [6,7], have constantly appeared. However, limited by their operating principle, these gyroscopes encounter difficulties in reaching their inertial grade [8]. In recent years, the disk resonator gyroscope (DRG) design has attracted increasing attention from researchers for its potential to achieve higher performance [9]. The DRG, which includes compact planar rings with central support and a distributed electrode, was inspired by hemispherical resonators [10]. Similar to the hemispherical resonator, the DRG can benefit from a high quality factor (Q), including a higher signal-to-noise ratio (SNR), better zero-bias stability, and lower power consumption [11]. Thus, a high Q is vital for a higher-performance DRG [12]. 

Q is a fundamental characteristic of a resonance system, which is defined by the ratio of the energy stored to the energy loss per unit time. The primary energy loss mechanisms of the resonator are thermoelastic damping, anchor loss, air damping, and surface loss [13]. Thermoelastic damping is caused by the temperature variation of the vibratory thin beams, which probably determine the Q of the vacuum packaged resonator [14]. The anchor loss is characterized by the energy transfer from the anchor to the substrate, which is affected by structure design [15] and fabrication imperfections [16]. Air damping is caused by the resistive force of the surrounding air but becomes negligible under the pressure of 0.1 mTorr [17]. Surface loss is the mechanical energy loss caused by surface defects, including dangling bonds, dust, and crystal termination defects, and it hardly plays a dominant role in MEMS resonators [18,19]. The four mechanisms determine the Q of the resonator itself, which is called $Q_{Unload}$. When the resonator is measured by an electric circuit, an additional damping related to experimental electronics is added to the resonance system [20]. Thermoelastic damping and anchor loss are more significant than air damping and surface loss. Thus, the Q for a high vacuumed MEMS resonator is

(1) $$\frac{1}{Q_{Total}}=\frac{1}{Q_{Unload}}+\frac{1}{Q_{Electronic}}\approx \frac{1}{Q_{TED}}+\frac{1}{Q_{Anchor}}+\frac{1}{Q_{Electronic}}.$$

The energy loss mechanism of thermoelastic damping is relatively mature and has been used to analyse the DRG [21,22,23]. However, the anchor loss of DRGs, especially considering their structurally asymmetry and fabrication imperfections, lacks careful study. Moreover, electronic damping has always been neglected, although the energy loss mechanism may affect the Q of the entire resonance system.

In this paper, the energy loss mechanism, including thermoelastic damping, anchor loss, and electronic damping, is performed based on our DRG design. More precisely, the effect of structural asymmetry and fabrication imperfections on anchor loss is analysed, and the electronic damping of the resonance system is deduced. Furthermore, the DRG is fabricated and measured to compare it with the theoretical result. This paper is organized as follows. Section 2 provides the design parameters and operating principles of the DRG. Section 3 presents the energy loss mechanism of the DRG, which is the main part of this paper. Section 4 illustrates the fabrication process and circuit testing results. The concluding remarks are summarized in Section 5.

## 2. Design and Operation

In this paper, the designed DRG consists of multiple nested rings as the sensing element, a single central anchor to fix the rings on the substrate, and embedded electrodes for the driving and sensing of the gyroscope. The two adjacent rings are connected by spokes, which offer a better vibration mode. Unlike the common equally spaced design, the nested loops used in this study are designed with unequal spacings. The electrodes are embedded in two rings, which are connected by long spokes, and the short spokes are used to ensure equal etching widths in the Deep Reactive Ion Etching (DRIE). Thus, the layout area can be more effectively used, and sufficient capacitance can be ensured while reducing the overall size of the structure.

**Figure 1:**
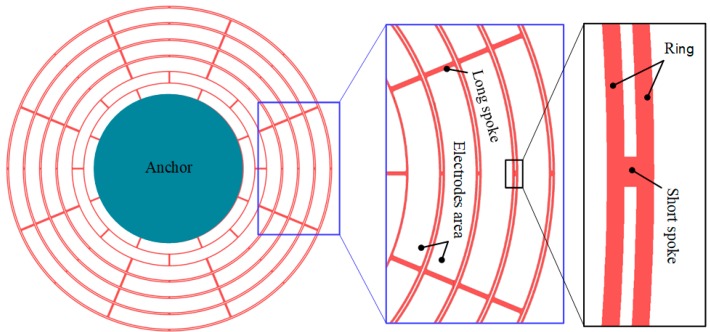
Geometry of the disk resonator gyroscope (DRG).

The geometry of the designed DRG is shown in Figure 1, where the embedded electrodes are removed for clarity. The designed DRG consists of 10 nested rings and 4 sets of embedded electrodes. The primary design parameters of the designed DRG are listed in Table 1. The long spoke and short spoke are 255 μm and 12 μm long, respectively, and the diameter of the outermost ring is 6000 μm. The 10 nested rings are suspended on a central anchor with a diameter of 2740 μm.

**Figure 2:**
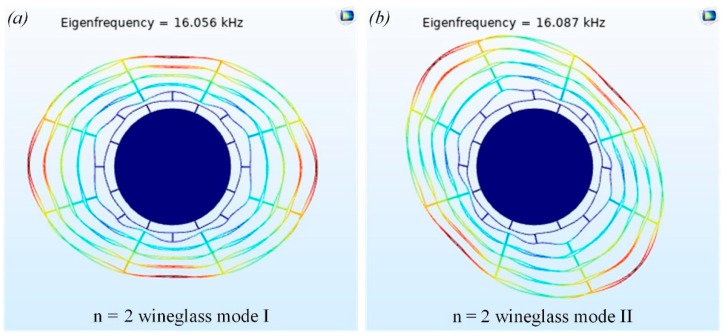
The n = 2 wineglass mode I (**a**) and the n = 2 wineglass mode II (**b**).

The DRG typically works in the n = 2 wineglass mode [24]. A finite element analysis was performed using COMSOL Multiphysics to calculate the frequency of the n = 2 wineglass mode. As shown in Figure 2, the two n = 2 wineglass modes, including the n = 2 wineglass mode I and n = 2 wineglass mode II, are approximately 16 kHz and 45° apart. In this paper, the n = 2 wineglass mode I was selected as the driving mode, and the n = 2 wineglass mode II was the sensing mode. 

## 3. Energy Loss Analysis

### 3.1. Thermoelastic Damping

The thermally isolated elastic structure produces a temperature variation when it suffers pressure or tension. To be precise, the temperature of the elastic structure decreases when it is uniformly stretched. The drop-in temperature is balanced by the increase in entropy, which is caused by the stress (since the process is reversible, the energy remains constant). Similarly, in compression, the elastic structure heats up. Under such ideal conditions, there is no energy loss, which implies no thermoelastic damping. However, in the real case, the elastic structure is always in a more complex normal mode, so there are regions of compression and extension. Depending on the timescale of the vibration, heat flows from the warmer parts of the structure to the cooler parts. Since the heat flow is an irreversible process, this heat flow is associated with the energy loss from the vibrational mode and the corresponding damping for the resonant mode.

Thermoelastic damping is particularly important for MEMS resonators, where regions of compression and expansion simultaneously appear. Zener [25,26] was the first to develop the theory of thermoelastic damping for thin rectangular beams under flexural vibrations, and the thermoelastic damping is [21]

(2) $$Q_{TED}^{-1}=\Delta_{M}\frac{\omega_{0}\tau }{1+{\left(\omega_{0}\tau \right)}^{2}}$$

where $\Delta_{M}$ is the relaxation strength, which only depends on the temperature and material properties and has a value of $2.02\times 10^{-4}$ for crystalline silicon at 298 K [27]. $\omega_{0}$ is the angular frequency of resonators. τ is the effective relaxation time and can be found in

(3) $$\tau =\frac{b^{2}\rho C_{p}}{\pi^{2}k}$$

where b is the thickness of the beam in the bending direction, ρ is the density of the material, $C_{p}$ is the heat capacity, and k is the thermal conductivity.

The key in the low thermoelastic damping design is to maintain the resonator away from the Debye peak [21], where $\tau \approx \omega_{0}{}^{-1}$. For the elastic beam with a width of 18 μm, the Debye peak appears at 467 kHz with $Q_{TED}$ of 9.5 k. Thus, the working frequency of the proposed DRG, which is 16 kHz, is far away from the Debye peak, and the corresponding $Q_{TED}$ is 139 k.

**Figure 3:**
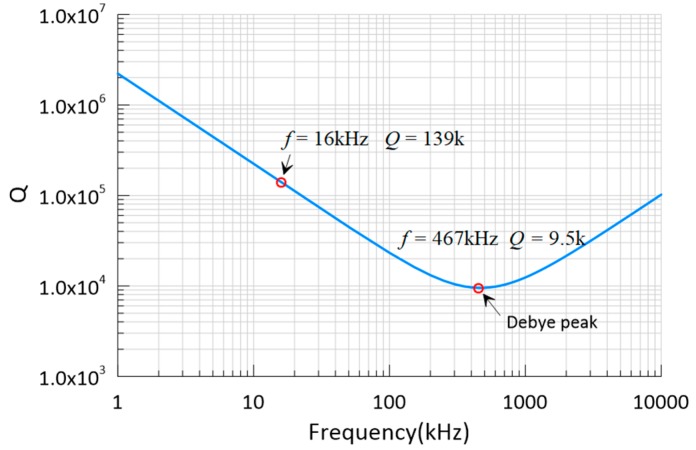
$Q_{TED}$ of an 18 μm wide beam at different frequencies.

**Figure 4:**
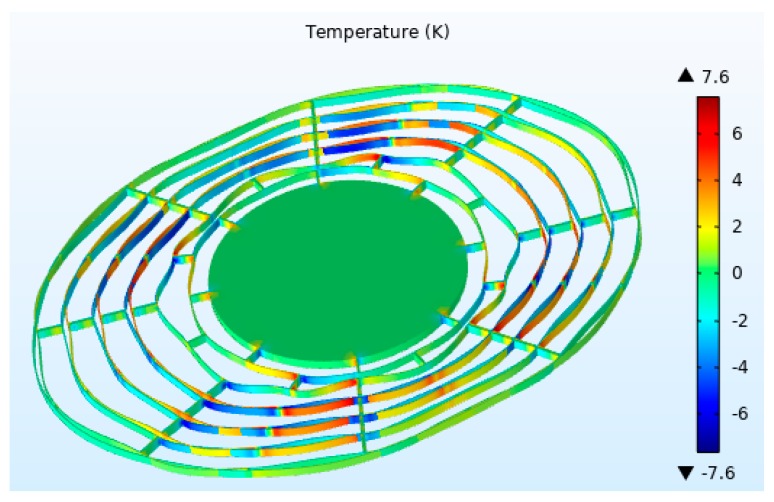
Temperature variation with respect to the reference temperature (298 K) for the n = 2 wineglass mode of the vibrating ring.

The thermoelastic damping shown in Figure 3 is for uniform beams under flexural vibrations. It has a deviation to describe the ring structures connected by long spokes and short spokes for DRG. In this paper, the finite element method is adopted to calculate the thermoelastic damping of DRG precisely. The thermoelastic damping of our DRG at 298 K is estimated in COMSOL (Version 5.4). Figure 4 shows the temperature variation reference of 298 K for the n = 2 wineglass mode, and the corresponding quality factor is 162 k. 

### 3.2. Anchor Loss

Anchor loss is a form of energy loss where the stress waves propagate away from the resonant structure, through the anchors and into the substrate. The anchor loss is affected by the configuration of the resonant structure and its operating condition. A symmetrical structure that operated in the anti-phase vibratory mode tends to reduce the energy dissipation for the minimized net reaction force applied to the substrate. These design tactics have been used and verified in the tuning fork gyroscope [11]. The DRG is fixed to the substrate by the central anchor, which has an inherent structure symmetry in design. Under ideal conditions, the net reaction force from the vibrating ring to the substrate at n = 2 wineglass modes is zero. Thus, the anchor loss is mainly caused by the inner stress that arises from the anchor deformation. However, the vibrating ring is commonly not perfectly symmetrical because of a fabrication error, which implies that the net force is not zero. Thus, apart from the inner stress, the external net force can also contribute to the anchor loss.

**Figure 5:**
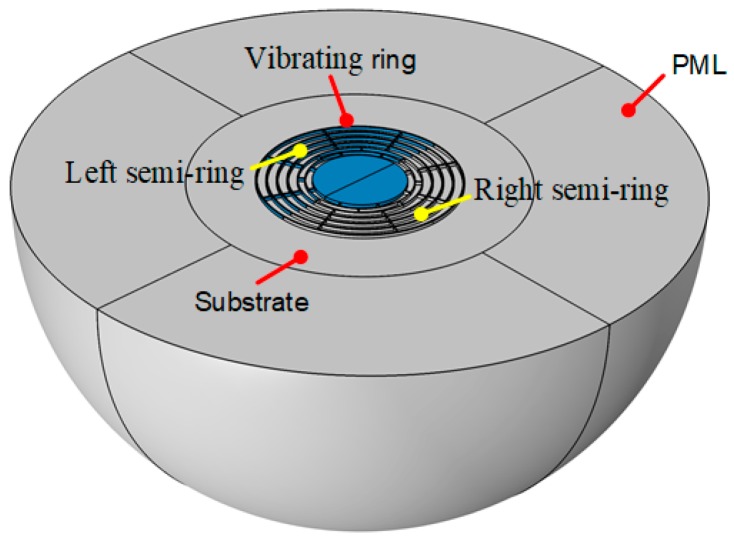
Anchor loss model for the DRG.

The anchor loss, i.e., the energy diffused into the substrate, can be calculated by the perfectly matched layer (PML) [28]. As schematically shown in Figure 5, the anchor loss model for the DRG is built in COMSOL. The hemispheric substrate is embraced by the hemispheric PML. Thus, the waves propagating away from the anchor are absorbed by the PML. The material of the vibrating ring is set as silicon, and the material of the PML and the substrate is set as borosilicate glass.

Previous research indicates that anchor loss highly depends on the thickness, inner radius, mesh quality, and scaling factor of the PML [29]. In this paper, the width of the PML is set as 0.56 m, which is equal to one wavelength, and the inner radius is equal to two wavelengths. The PML is swept with 12 nodes, and the scale factor sweeps in the range of $10^{-4}$ to $10^{4}$. Using the method introduced in [30,31], a Q factor of 130 million is acquired.

The calculated Q factor is mainly contributed by the anchor deformation. However, the asymmetry of the vibrating ring caused by fabrication errors should also be considered. In this paper, the stiffness and mass asymmetry of the rings are considered. The asymmetry of stiffness and mass is caused by fabrication imperfections and manifests as a structural dimension mismatch. Building models with variational structural dimensions is infeasible in COMSOL. Thus, the material’s densities and Young’s modulus are picked to simulate mass and stiffness asymmetry. In the mass asymmetry model, the density of the right semi-ring is set as 2330×(1−α) kg/m^3^, where α ranges from 0.1% to 10%. Stiffness asymmetry is simulated by changing the Young’s modulus of the right semi-ring. The material parameters of the two models are given in Table 2.

**Figure 6:**
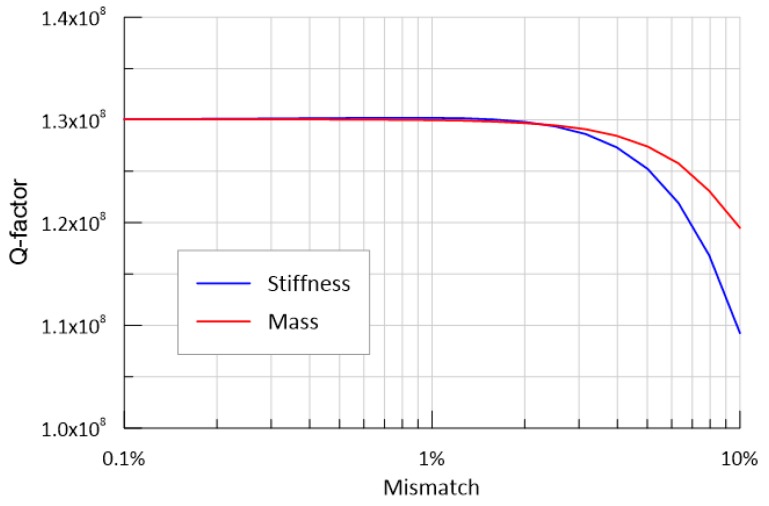
Effect of the stiffness and mass mismatch on the Q factor.

Figure 6 shows the Q factor variation when the mismatch changes from 0.1% to 10%. The stiffness asymmetry has a greater effect on Q than the mass mismatch. The stiffness and mass asymmetry have a negligible effect on the Q factor for their mismatch within 1%. However, the Q factor begins to markedly decrease when the mass mismatch exceeds 1%. For our gyroscope, the stiffness and mass mismatch between two semi-rings is believed to be within 1%. Thus, the Q factor of 130 million can be used to estimate the total anchor loss.

### 3.3. Electronics Damping

**Figure 7:**
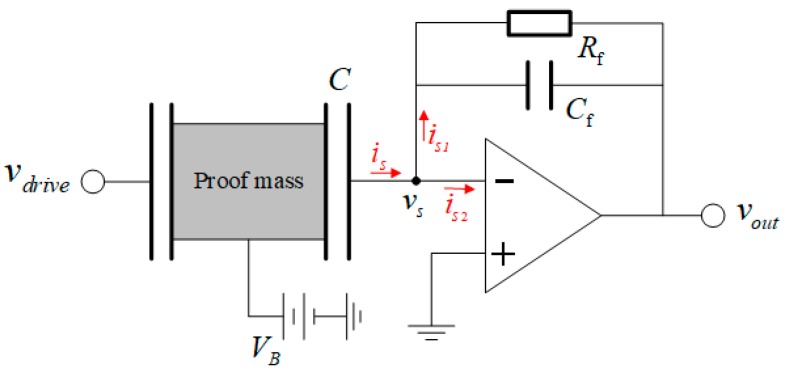
Electrical circuit model.

The DRG was excited by applying an AC voltage to the outer capacitor, and the sense signal from the sensing capacitor is amplified using a simple amplification circuit. At the output, the required voltage bias to generate a readout signal can also place a force on the sensor and cause a damping, which is called electronics damping. Unlike the aforementioned energy loss mechanisms, the electronics damping is not associated with the gyroscope design and fabrication but relates to experimental electronics [32]. The electrical circuit model of the experimental setup is shown in Figure 7. Thus, the DRG can be described using the governing equation of a second-order mechanical system with the electrostatic forces from the two electrodes as follows:

(4) $$m\ddot{x}+c\dot{x}+kx=F_{v_{drive}}+\frac{{\left(V_{B}-v_{s}\right)}^{2}}{2}\frac{dC}{dx}$$

where *m*, *k*, and *c* are the modal mass, modal stiffness, and damping of the DRG, $F_{v_{drive}}$ is the force generated by the driving signal, and $\frac{{\left(V_{B}-v_{s}\right)}^{2}}{2}\frac{dC}{dx}$ is the parasitic force generated by the sensing signal.

For an ideal operation amplifier (op-amp), the amplifier gain is infinite, and the node voltage is equal to the noninverting input. However, in reality, the amplifier gain is finite, and the sensing signal can be analysed as follows:

(5) $$i_{s}=i_{s1}+i_{s2}$$

(6) $$i_{s}=\left(V_{B}-v_{s}\right)\frac{dC}{dt}$$

(7) $$i_{s1}=\frac{v_{s}+Av_{s}}{Z_{f}}$$

(8) $$i_{s2}=\frac{v_{s}}{Z_{in}}.$$

Then, substituting Equations (6)–(8) into Equation (5), and using Taylor’s series expansion, we obtain the following relation:

(9) $$V_{B}-v_{s}=V_{B}(1-\alpha \frac{dC}{dt})$$

(10) $$\alpha =1/(\frac{A+1}{Z_{f}}+\frac{1}{Z_{in}})$$

where $Z_{in}$ is the input impedance of the op-amp, A is the amplifier gain, and $Z_{f}$ is the feedback impedance.

Considering that

(11) $$\frac{dC}{dt}=\frac{dC}{dx}(\frac{dx}{dt})=\frac{dC}{dx}\dot{x}$$

Then, substituting Equation (9) into Equation (4), we can write:

(12) $$m\ddot{x}+c\dot{x}+kx=F_{v_{drive}}+\frac{1}{2}V_{B}^{2}\frac{dC}{dx}-\alpha V_{B}^{2}{\left(\frac{dC}{dx}\right)}^{2}\dot{x}+\frac{1}{2}\alpha^{2}V_{B}^{2}{\left(\frac{\partial C}{\partial x}\right)}^{3}{\dot{x}}^{2}.$$

The third term on the right side of Equation (11) is the damping term due to the experimental electronics, since it is proportional to the velocity. According to the fundamental relation between the damping and Q of a mechanical system, c=mω/Q, and Q from the experimental electronics is calculated as:

(13) $$Q_{electronics}=\frac{m\omega }{V_{B}^{2}{\left(dC/\mathrm{dx}\right)}^{2}\alpha }.$$

**Figure 8:**
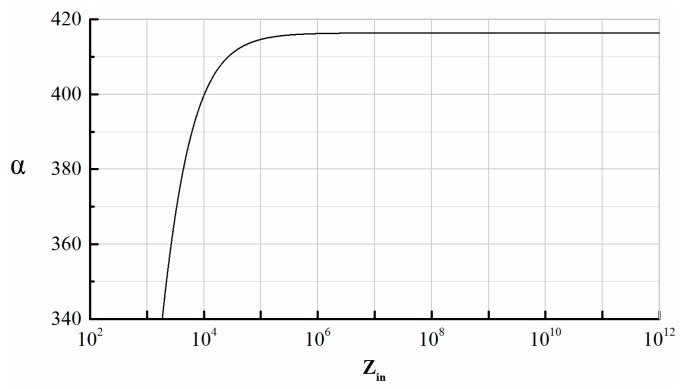
Relationship between α and $Z_{in}$.

The op-amp in our experiment is AD8065 with an amplifier gain of 113 dB. The differential input impedance *Z_in_* of AD8065 is 1000 GΩ//4.5 pF, which is equal to 2.2 MΩ at 16 kHz. As illustrated in Equations (10) and (12), *Z_in_* can contribute electronic damping through the intermediate variable α. The variable α starts to change significantly only when *|Z_in_*| is below $10^{5}$ (Figure 8), so the amount of electronic damping remains approximately constant for *|Z_in_*| > $10^{5}$.

Using the mechanical and electrical parameters of the proposed DRG, as listed in Table 3, and Equation (12), we can write:

(14) $$Q_{electronics}=6.09\times 10^{8}/V_{B}^{2}.$$

**Figure 9:**
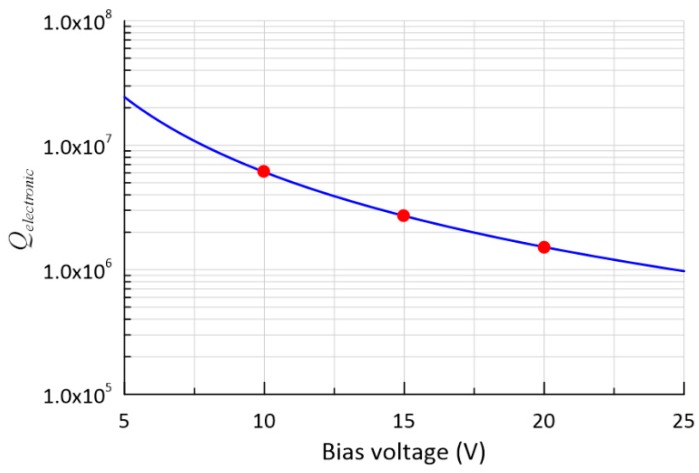
Relationship between Q and the bias voltage.

Thus, the electronic damping is proportional to the square of the bias voltage ($V_{B}$). The relationship between the electronic damping and the bias voltage is shown in Figure 9, where $Q_{electronics}$ is 6.09 million, 2.71 million, and 1.52 million, with bias voltages of 10 V, 15 V, and 20 V, respectively. 

### 3.4. Q Calculation of the DRG

The calculated Q induced by thermoelastic damping, anchor loss, and electronic damping is summarized in Table 4. There are orders of magnitude of difference among the three energy loss mechanisms. The thermoelastic damping has a contribution of over 90%, which plays a dominant role in the energy loss of the DRG. Benefiting from the symmetrical structure, the anchor loss is low (~0.1%) and can be neglected. The electronic damping determined by the testing circuit contributes 2.6%–9.6% when the bias voltage increases from 10 V to 20 V, which considerably affects the total Q.

## 4. Verification and Discussion

**Figure 10:**
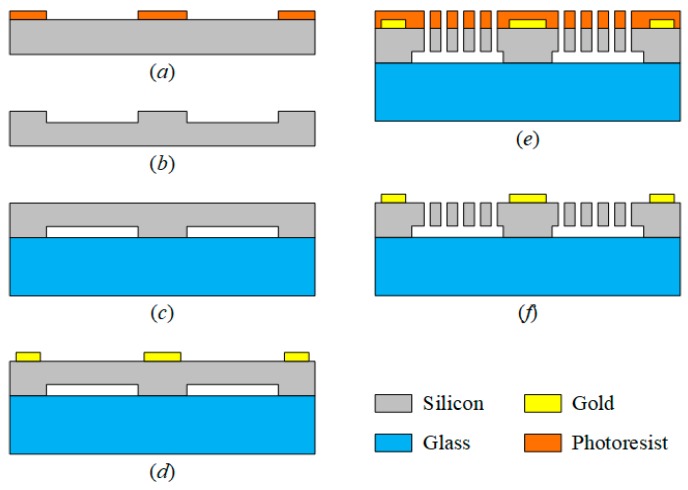
Fabrication process.

The DRG is fabricated by SOG (silicon on glass) instead of SOI (silicon on insulator) technology for a more flexible process. The fabrication process is demonstrated in Figure 10. The process starts by forming the photoresist mask on a silicon wafer with a thickness of 300 μm (Figure 10a). Then, the wafer is etched 50 μm deep by deep reactive ion etching (DRIE) to form bonding bumps (Figure 10b). Third, the patterned wafer is bonded with a borosilicate glass of 500 μm, which thins the silicon wafer to 100 μm (Figure 10c). Fourth, a 200 nm gold film is sputtered on the bonded wafer and patterned (Figure 10d). Then, the bonded wafer is coated with photoresist and patterned by DRIE to form a vibrating ring structure (Figure 10e). By removing the photoresist, we obtained the DRG chip (Figure 10f). 

**Figure 11:**
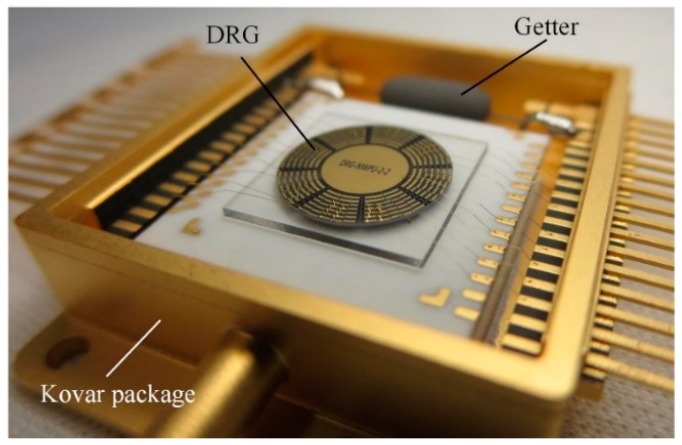
DRG in a Kovar package with the getter inside.

As shown in Figure 11, the fabricated gyroscope is vacuum packaged in a Kovar package with the getter inside. Previous experiments indicate that the air pressure in the sealed package is under 0.01 Pa. Thus, the air damping of the gyroscope is not the dominant energy loss type compared to other energy loss mechanisms.

**Figure 12:**
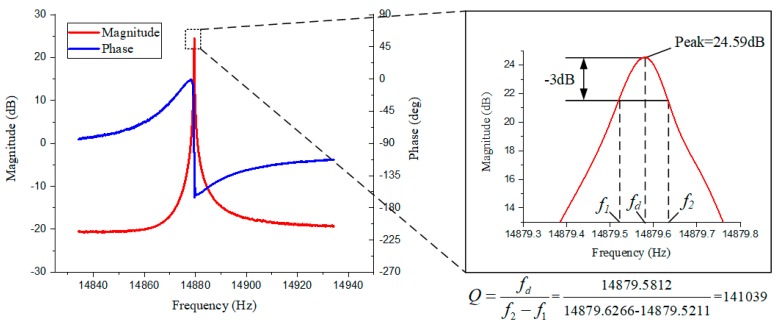
The resonance peak and phase of the DRG 5 with the highest Q.

The sealed gyroscopes were tested using the electrical circuit in the electronics damping part. Five chips were tested, and the results are listed in Table 5. The frequency of the gyroscopes was 14.8–14.9 kHz, which is smaller than the design value of 16 kHz. The Q of the gyroscope was 71.4–141 k when the bias voltage was equal to 10 V. The Q of DRG 5 was further measured with different bias voltages, and the result is shown in Table 6. The Q of DRG 5 shows a significant decrease when the bias voltage increases, which is consistent with the theoretical analysis. The resonance peak and phase of DRG 5 with the highest Q is show in Figure 12.

In addition, the measured maximum Q value has a relative deviation of approximately 10% compared to the theoretical model. There are several potential reasons for this deviation:

- The fabrication error introduces a dimensional variation to the DRG structure, which affects the theoretical results (especially those for the thermoelastic damping). This is the most likely reason.
- There is a readout error in measuring Q using the half-power bandwidth method, although we have done our best to avoid this error.
- The air damping and surface loss contribute to Q.

## 5. Conclusions

The thermoelastic damping, anchor loss, and electronic damping of the proposed DRG are calculated. The results show that thermoelastic damping is the dominant energy loss mechanism, with a contribution of over 90% of the total dissipated energy, the anchor loss is negligible, and the electronic damping contributes within 10%. In theory, the Q of the proposed gyroscope has a difference of approximately 10% of the test results. The potential reasons for this difference may be due to a fabrication error, a measuring error, or other loss mechanisms. In general, the thermoelastic damping and electronic damping essentially determine the Q of the DRG. Moreover, optimizing the resonance structure and testing circuits to reduce thermoelastic damping is important for high-performance DRG design.

## Figures and Tables

**Table 1:** Geometrical parameters of the DRG.

Parameters	Value	Parameters	Value
Diameter of the outermost ring	6000 μm	Width of rings	18 μm
Diameter of the innermost ring	2740 μm	Thickness of rings	100 μm
Angle between short spoke	45°	Width of short spoke	30 μm
Angle between long spoke	45°	Length of short spoke	12 μm
Angle between short and long spoke	22.5°	Width of long spoke	40 μm
Number of rings	10	Length of long spoke	255 μm

**Table 2:** The material parameters in mass asymmetry and Young’s modulus asymmetry models.

Items	Mass Asymmetry Model		Stiffness Asymmetry Model	
	*ρ* (kg/m^3^)	E (GPa)	*ρ* (kg/m^3^)	E (GPa)
Left semi-ring	2330	169	2330	169
Right semi-ring	2330 × (1−α)	169	2330	169 × (1−α)

**Table 3:** System parameter of the DRG.

Parameter	Value
Proof mass (m)	$7.27\times 10^{-7}$ kg
Angular frequency (ω)	$1.01\times 10^{5}$ rad/s
Change rate (dC/dx)	$5.36\times 10^{-7}$ F/m
Feedback impedance ($\left|Z_{f}\right|$)	186 MΩ

**Table 4:** Calculated Q of the DRG.

Item	Q	Contribution
$Q_{TED}$	$1.62\times 10^{5}$	~97.5%
$Q_{Anchor}$	$1.3\times 10^{8}$	~0.1%
$Q_{electronics}$	$6.09\times 10^{6}\;(V_{B}=10V)$ $2.71\times 10^{6}\;(V_{B}=15V)$ $1.52\times 10^{6}\;(V_{B}=20V)$	~2.6% ~5.5% ~9.6%
$Q_{Total}$	$1.58\times 10^{5}\;(V_{B}=10V)$ $1.53\times 10^{5}\;(V_{B}=15V)$ $1.46\times 10^{5}\;(V_{B}=20V)$	

**Table 5:** Frequency and Q of the fabricated DRGs.

No.	Frequency (kHz)	Q (Bias Voltage 10 V)
DRG 1	14.8	71.4 k
DRG 2	14.9	86.9 k
DRG 3	14.8	108 k
DRG 4	14.9	109 k
DRG 5	14.9	141 k

**Table 6:** Q of the DRG 5 with different bias voltages.

Bias Voltage (V)	10	15	20
Q	141 K	137 K	132 K
